# Notched Cartilage With Incus Interposition Ossiculoplasty Combined With Cavity Obliteration: A Novel Approach: Analysis of Hearing Results in 50 Cases

**DOI:** 10.1097/ONO.0000000000000095

**Published:** 2026-08-11

**Authors:** Sekhar Bandyopadhyay, Arijit Das

**Affiliations:** 1 Department of Otolaryngology & Head and Neck Surgery, North Bengal Medical College & Hospital, Darjeeling, India.

**Keywords:** Cavity obliteration, Incus interposition, Notched cartilage, Ossiculoplasty

## Abstract

**Background:**

In chronic suppurative ear disease, conductive hearing loss with ossicular chain erosion, poses a serious challenge to the otologist. To overcome this hurdle, various ossiculoplasty techniques have been developed to date. Little modifications in the ossicular reconstruction technique, combined with cavity obliteration in selected cases, can yield predictably good results.

**Objective:**

A) To study the hearing outcome in 50 patients of Austin group A ossicular defect, using a piece of notched autologous conchal cartilage over the stapes suprastructure, combined with incus interposition and cavity obliteration. B) To compare the results of hearing outcomes of the present study with other methods of ossiculoplasty.

**Methods:**

The present study includes patients who underwent ossiculoplasty for Austin classification group A ossicular defect (M+, S+, and eroded long process of incus) with radiologic evidence of bone erosion involving air cells in the tympanomastoid region, and patients with tubotympanic (safe) disease having peroperative findings of massive irreversible mucosal disease involving the mastoid antrum and aditus. The study was done at North Bengal Medical College Hospital, Darjeeling, between November 1, 2017 and October 31, 2021. Preoperative high resolution computed tomography temporal bone, pure tone audiometry (preoperative and postoperative), and histopathological examination of the diseased tissue were done in all cases. Canal wall down mastoidectomy was done in all cases. A small piece of conchal cartilage was harvested (thickness: 0.5 mm, diameter: 5 mm). A central notch was fashioned with a 0.6 mm diamond burr to accommodate the head of the stapes. The body of the incus was placed between the notched cartilage and the malleus head. Temporalis fascia was harvested and placed between the anterior part of the annulus and the mastoid cavity, covering the neo-ossicular chain. Two flaps (superiorly based and inferiorly based) with periosteum and soft tissue were used for cavity obliteration.

**Results:**

n = 50, male 26, female 24. Atticoantral disease: 32 cases, tubotympanic disease:18 cases. Preoperative average air-bone (AB) gap: 29.3 dB, postoperative average AB gap: 19.7 dB. Significant improvement in postoperative AB gap (mean: 19.7 dB) in comparison with preoperative AB gap (mean: 29.3 dB) with a *P* < 0.001 was observed in 74% of patients with notched cartilage and incus interposition ossiculoplasty and cavity obliteration.

**Conclusion:**

Seventy-four percent of ossiculoplasty patients achieved a postoperative AB gap within 20 dB. This technique of ossiculoplasty with autologous cartilage and ossicle with cavity obliteration is physiological, biocompatible, and stable.

In chronic suppurative ear disease management, reconstruction of the ossicular chain defect is of paramount importance. Due to its precarious blood supply, the long process of the incus is susceptible to osteitic resorption. During surgery for suppurative ear disease, minor erosion of the lenticular process and more pronounced resorption of the long process are common findings. As per the Austin classification of ossicular chain defects with absent incus, there are 4 groups: (malleus, incus, and stapes are indicated by their initials and their presence and absence by + and − respectively); (Table 1). In chronic suppurative ear disease, group A (M+ S+ I−) constitutes 60% of all ossicular defects, followed by group B (M+ S− I−): 23%, group C (M− S+ I−): 8%, and group D (M− S− I−): 8% (1).

**TABLE 1.** Austin classification of ossicular chain defects with absent incus

Group	Ossicular status	Abbreviations	Percentage (%)
A	Malleus handle and stapes suprastructure present	M+ S+	60
B	Malleus handle present and stapes suprastructure absent	M+ S −	23
C	Malleus handle absent and stapes suprastructure present	M− S +	8
D	Malleus handle and stapes suprastructure absent	M− S−	8

Zollner (2) first described the correction of ossicular defects by ossiculoplasty. Since then, several methods have evolved in ossiculoplasty with different techniques and different graft materials, including allografts, autologous and homologous cartilages, and prosthetics like titanium, gold, Teflon, and hydroxyapatite. The results of ossicular reconstruction depend on different prognostic factors, surgical technique, graft material, type of prosthesis, and experience of the surgeon.

## MATERIALS AND METHODS

The present study includes a series of fifty (50) patients who underwent ossiculoplasty for Austin group A ossicular defects (M+, S+ I−, eroded long process of incus) at North Bengal Medical College Hospital, Darjeeling, over 4 years. All the patients with chronic suppurative ear disease, with radiologic evidence of ossicular chain erosion (Austin classification group A) and conductive hearing loss with cholesteatoma (atticoantral), and patients with tubotympanic disease having radiologic and peroperative findings of irreversible mucosal disease of the tympanomastoid region, were included in the study. Patients with Austin group B, C, D, and those with sensorineural or mixed hearing loss were excluded from the study. All the ossicular reconstructions were performed in a tertiary care center in eastern India by the senior author between November 1, 2017 and October 31, 2021.

Preoperative high resolution computed tomography (HRCT) temporal bone (0.6 mm cuts), pure tone audiometry (preoperative and postoperative), examination under a microscope, and histopathology examination of the diseased tissue were done in all cases at the same institute. The first postoperative pure tone audiometry was done after 6 months of the operation and every 6 months thereafter until the last follow-up.

To comply with Level I of the Committee on Hearing and Equilibrium guidelines of the American Academy of Otolaryngology and Head and Neck Surgery released in 1995, the Pure Tone Average was calculated as the mean of the 0.5, 1, 2, and 4 kHz thresholds. Air-bone gap (ABG) was calculated from air conduction and bone conduction thresholds determined in each study. Postoperative hearing gain was calculated from the Pure Tone Average before the surgery and at the last follow-up examination. The mean follow-up period was 19.2 months (range between 6 and 78 months). Since the word recognition score was not included in the study, the standardized format of reporting the hearing outcome in the form of a scatterogram could not be followed. Only preoperative and postoperative pure tone audiograms were studied, and the results were analyzed as per conventional norms in the form of ABG.

An informed consent form was signed by all patients regarding the publication of data from the study under the condition of anonymity.

Statistical analysis was performed with the Jamovi software version 2.4.1. The statistical significance of the differences in means of data was determined using the paired sample Student’s *t*-test for comparing results in the same set of cases. A significance level of 0.05 (2-tailed) was assumed for all the calculations to determine whether to reject the null hypothesis.

## SURGICAL TECHNIQUE

**FIG. 1.**
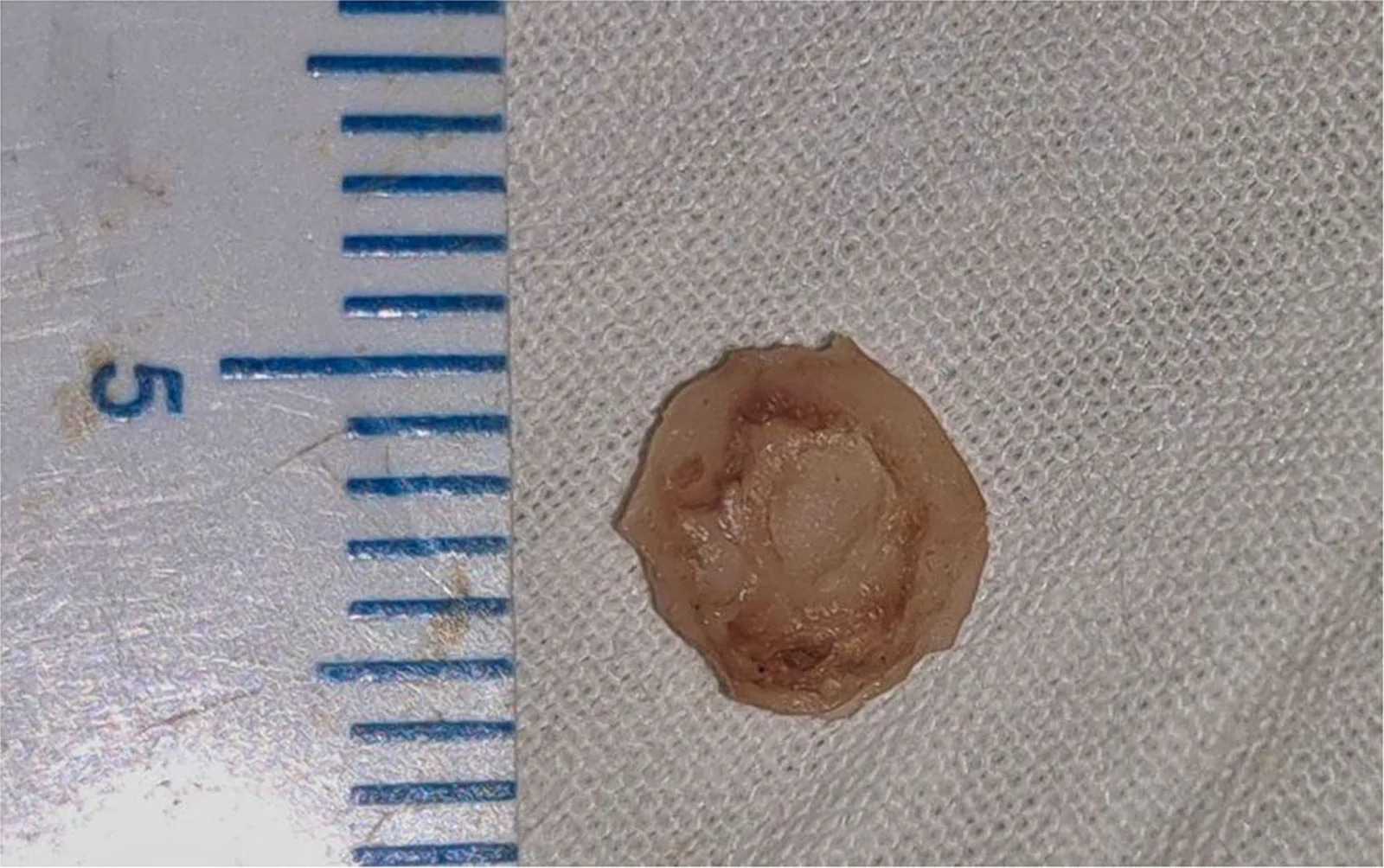
Notched cartilage. A central notch is fashioned in the conchal cartilage (diameter 5 mm [approx.], thickness: 0.5 mm [approx.]).

Canal wall down mastoidectomy with tympanoplasty was done in all cases. A small piece of conchal cartilage was harvested (thickness: 0.5 mm, diameter: 5 mm). A central notch was fashioned in this cartilage with a 0.6 mm diamond burr to accommodate the head of the stapes (Fig. 1). After placement of the notched cartilage over the head of the stapes, the body of the incus was placed over the notched cartilage.

**FIG. 2.**
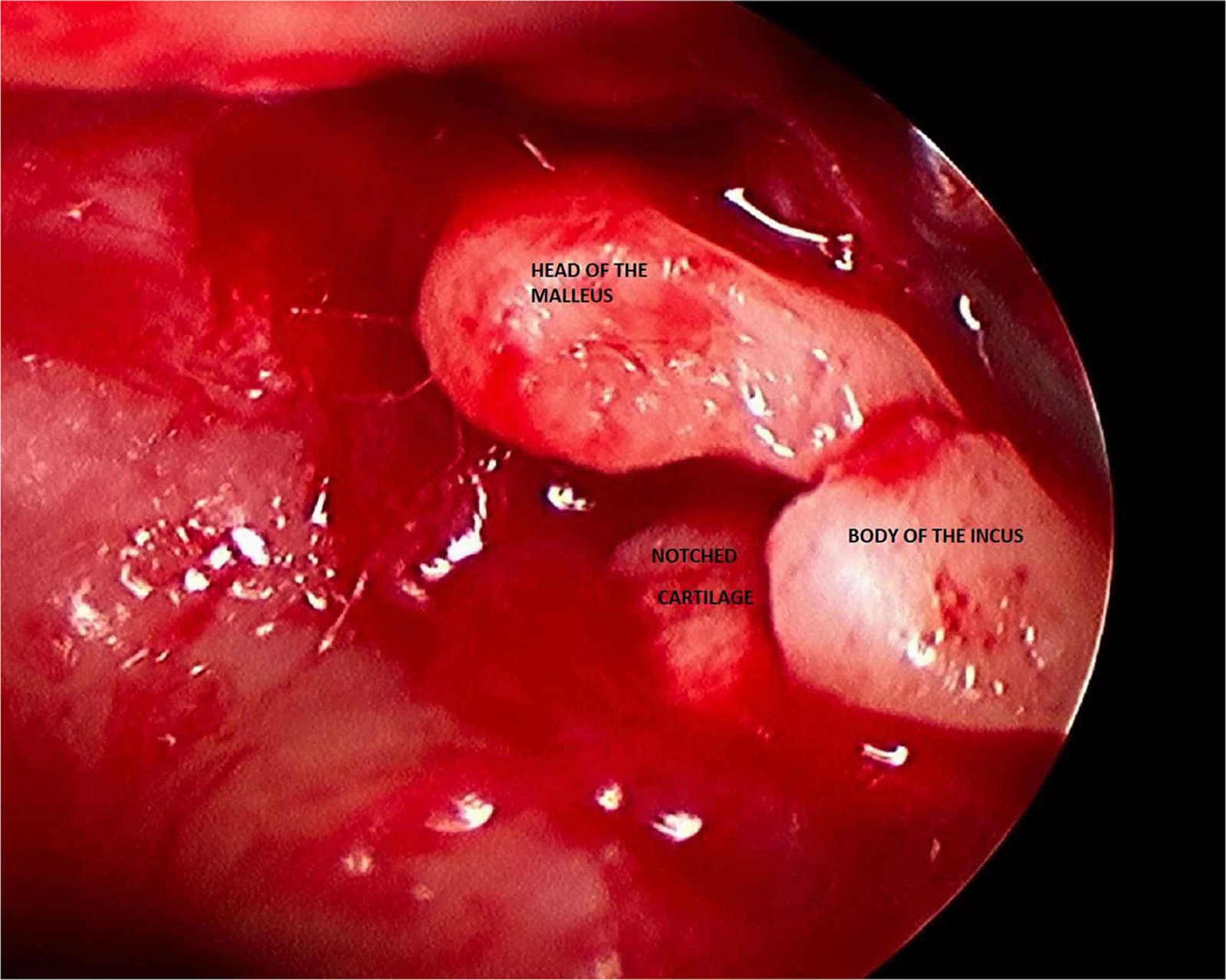
Ossicular reconstruction just before cavity obliteration. INC indicates body of the incus; N.C, notched cartilage; TFG, temporalis fascia graft.

The tensor tympani, with its attachment to the malleus handle along with the malleus head, was also preserved in all the cases in this series (3). A near-normal catenary lever mechanism (1) was restored by snugly fitting the notched cartilage over the head of the stapes, placed medial to the head of the malleus and reinforced by the interposition of the body of the incus over the cartilage (Fig. 2).

Temporalis fascia was harvested and placed between the anterior part of the annulus (under the drum remnant) and the mastoid cavity, covering the neo-ossicular chain. Two flaps, superiorly and inferiorly based, composed of soft tissue and periosteum, were used for cavity obliteration.

## RESULTS

Fifty patients (n = 50) underwent notched cartilage ossiculoplasty in this series. There were 26 (52%) males and 24 (48%) female patients. The mean age of the patients was 26.7 years (range: between 8 and 55 years). Thirty-two (64%) patients had atticoantral disease, and 18 (36%) had tubotympanic disease. Eight patients had dural or sinus plate erosion. Two patients had facial canal erosion, of which 1 patient had House Brackmann grade V facial palsy, who recovered completely after facial nerve decompression in the same sitting with ossiculoplasty.

On histopathological examination of the diseased tissue, 13 (26%) patients were found to have cholesteatoma, while 37 (74%) had granulation tissue.

As per the American Academy of Head and Neck Surgery guidelines, a successful outcome is defined as closure of the postoperative ABG by ≤20 dB (4). In the present study, postoperative ABG < 20 dB was considered successful. The closure in ABG was calculated as the difference between pre- and post-operative ABG.

Preoperative Mean ABG was 29.4 dB (SD: ± 9.45 dB) while the postoperative average ABG was 19.7 dB (SD: ±10.28 dB) (Table 2). Paired sample Student’s *t* test shows the postoperative group had better hearing (mean ABG: 19.7 dB) than the preoperative group (mean ABG: 29.4 dB), with a significant reduction of 9.67 dB in ABG after operation (*P* < 0.001).

**TABLE 2.** A comparative literature review of related and present studies

	Present study	Castro Sousa et al. (2016) (5)	Iurato et al. (2001) (6)	Chavan et al. (2017) (7)	Dornhoffer (2003) (8)	Mundada and Jaiswal (1989) (9)
Sample size Mean age (years) M:F ratio Follow-up	n = 50 26.7 (range 8–55 years) 26:24 19.2 months	n = 153 – 85:68 –	n = 181 39 years 6 months 90:91 2 years 7 months (range: 1–16 years)	n = 50 36.4 years 29:21 –	n = 636 30 years (range: 4–88 years) – 2.7 years (range: 3–7 years)	n = 115 – – 1–13 years
Surgical technique	Notched cartilage + Incus interposition + Canal wall down Mastoidectomy with cavity obliteration	Autologous ossicle Cortical bone/cartilage titanium used as prosthesis	Autologous/homologous body of the incus, head of the malleus, PORP used as prosthesis	Autologous INCUS in 40 (80%) cases, head of the malleus in 10 (20%) cases–used as ossiculoplast	PORP: 252 cases TORP: 158 cases Mastoidectomy: 236 cases	Tunnel between the outer perichondrium and the cartilage to accommodate the remnants of the Incus or handle of the malleus
Inclusion criteria	Austin group A	All patients with ossicular discontinuity	Austin-Kartush group A		Three groups of patients: Cholesteatoma, atelectasis, high-risk recurrent perforation	_
Preoperative ABG (dB)	29.4 dB ± 9.45 dB SD	35.3 dB ± 10.6 dB SD	26 dB ± 12 dB SD	34.9 dB ± 10.15 dB SD	25.7 dB ± 11.8 dB SD	
Postoperative ABG (dB)	19.7 dB ± 10.28 dB SD	14 dB ± 10.5 dB SD	26 dB ± 12 dB SD	12.92 dB ± 7.92 dB SD	14.1 dB ± 9.5 dB SD	<20 dB in 74% of patients
*P*-value	<0.001	<0.05	Not significant	<0.001	<0.05	<0.01
Remarks	With cholesteatoma 13(26%) cases, without cholesteatoma 37 (74%) cases	With cholesteatoma 57 (37.1%) cases, without cholesteatoma-96 (63%) cases	Perforation of tympanic graft in 13 (7.2%) cases after ossiculoplasty. (patients with extensive cholesteatoma excluded)	Patients with cholesteatoma excluded	Complications: 14 (2.2%) perforations, 21 (3.3%) revision surgery, 8(1.3%) recurrent cholesteatoma, 3 (0.5%) delayed facial palsy, complete recovery within 4–6 weeks after operation	Cholesteatoma in 2(1.7%) cases, granulation in 26 (22.6%) cases, eroded INCUS in 73 (63.4%) cases

ABG indicates air-bone gap; PORP, partial ossicular reconstruction prosthesis; TORP, total ossicular reconstruction prosthesis.

Postoperative ABG closure < 20 dB was observed in 37 (74%) cases of notched cartilage ossiculoplasty with incus interposition.

Paired sample Student’s *t* test shows in patients aged between 20 and 39 years, postoperative mean ABG 20.1 dB was better than preoperative mean ABG 30.3 dB, with a significant reduction of 10.22 dB in ABG (*P* < 0.001). In patients aged more than 40 years there was postoperative mean ABG 17.5 dB. It was better than the preoperative mean ABG 26.3 dB, with a significant reduction of 8.75 dB in ABG after operation (*P* < 0.022). On average, for patients aged less than 20 years, postoperative mean ABG 19.6 dB was better than preoperative mean ABG 28.5 dB with a significant reduction. Although there is a reduction of 8.86 dB in ABG after operation, it is not statistically significant (*P* < 0.092).

Out of 13 cases of cholesteatoma, 8 (61.5%) patients had postoperative mean ABG:20.5 dB and preoperative mean ABG: 32.4 dB with a reduction of 11.9 dB in mean ABG after operation (*P* < 0.001).

Out of 37 patients with histopathology of granulation tissue, 29 (78.4%) patients had postoperative mean ABG: 19.4 dB and preoperative mean ABG: 28.3 dB with a reduction of 8.88 dB in mean ABG after operation (*P* < 0.001).

However, there was no statistically significant association between postoperative improvement in mean ABG and the histopathological type of the disease.

In male patients, there was postoperative mean ABG: 17.8 dB and preoperative mean ABG: 29.4 dB with a reduction of 11.6 dB in mean ABG after operation (*P* < 0.001). In female patients though, there was a postoperative mean ABG: 21.7 dB and a preoperative mean ABG: 29.3 dB with a reduction of 7.58 dB in mean ABG after operation (*P* < 0.028).

Patients with semicircular canal fistula and granulation around the footplate area had poor ABG closure.

Notably, there was no extrusion of either notched cartilage or the incus, presumably due to the use of autologous tissues.

## DISCUSSION

**FIG. 3.**
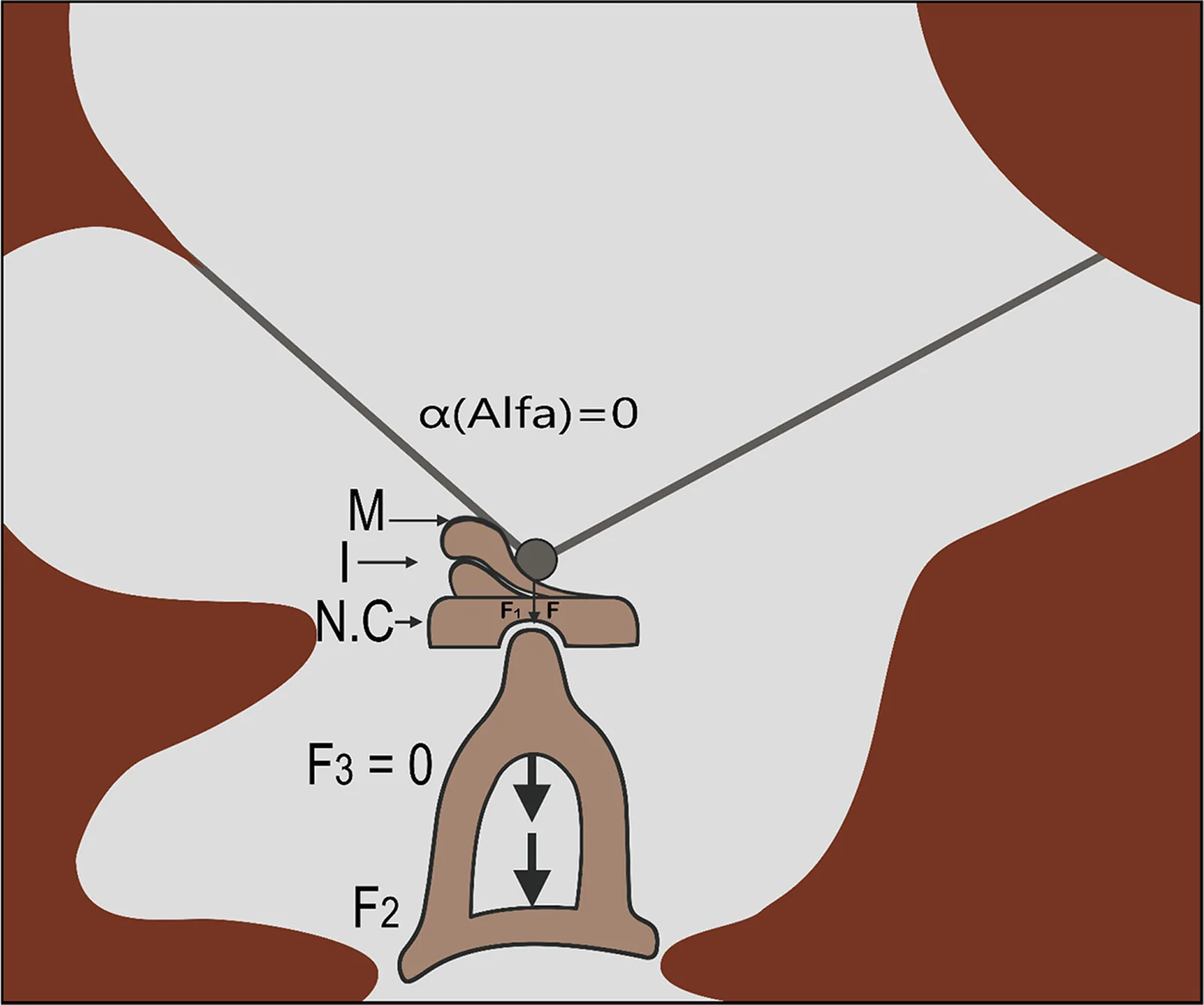
Principles of forces acting on neotympanum. α, angle between applied force and resultant force; F, force acting on the neotympanum; F1, resultant force; F2, force acting on the footplate; F3, tilting force; I, repositioned body of the incus; M, malleus; N.C., notched cartilage.

To an otologist, the challenge in surgery for chronic suppurative ear disease is to achieve a safe dry ear with hearing improvement. In Austin Group A patients (M+ S+ I−, ie, malleus handle and stapes are present and the long process of the incus is absent), the main goal is to establish a stable connection between the handle of the malleus, mobile stapes, and the tympanic membrane. Although the whole reconstructed ossicular chain and the neotympanum are not native, adhering to the basic principles of surgery should lead to restoration of a near-normal catenary lever mechanism with minimum tilting force (F3), optimum resultant force (F1), and force acting on the footplate (F2) (Fig. 3) (1).

In the largest series of 1000 patients of Cartilage Tympanoplasty, 636 patients were eligible for outcome analysis (8). Average preoperative ABG was 25.7 ± 11.8 dB SD, postoperative ABG was 14.1 ± 9.5 dB SD with a *P* < 0.05. Partial ossicular reconstruction prosthesis (PORP) was used in 252 cases, while total ossicular reconstruction prosthesis was used in 158 cases. Mastoidectomy was done in 236 (37%) cases. Cartilage was used as island flaps (in 98 cases of atelectatic ears) or palisade graft (in 220 cases of cholesteatoma) for tympanic membrane reconstruction only.

In a series of 153 patients (5) ossicular reconstruction was done with autologous ossicles, cortical bone, cartilage, and titanium. 57 (37%) patients had cholesteatoma in this series. Malleus handle was present in 70 (45.7%) cases, and stapes suprastructure present in 90 (58.8%) cases. 74% patients achieved postoperative mean ABG < 20 dB in this study.

Autologous or homologous body of the incus, head of the malleus and PORP were used for ossicular reconstruction in another series of 181 cases (6). Extensive cholesteatoma patients were excluded in this series. Limited cholesteatoma disease was present in 56 (31%) cases.

In a series of n = 115 cases (9), mastoidectomy with fashioning a tunnel between cartilage and the outer perichondrium to accommodate remnants of the incus or the handle of the malleus was done. Postoperative mean ABG < 20 dB was achieved by only 24.4% patients in this series. This is statistically insignificant.

Another series (n = 50) (7) reported use of autologous malleus and incus for ossicular reconstruction. Cholesteatoma patients were excluded from the study. Preoperative ABG was 34.95 ± 10.15 dB SD, and postoperative ABG was 12.92 ± 7.92 dB SD in this series. With a mean age of 36.4 years, 84% of patients achieved postoperative mean ABG < 20 dB. All 33 (66%) patients of Austin Kartush group A in this series achieved a postoperative mean ABG of 11.59 dB. The period of follow-up was not mentioned in the study.

In the present series, autologous notched cartilage was used over the mobile stapes suprastructure to minimize the tilting force (F3). This assembly was further reinforced by the body of the incus, bridging the gap between the handle of the malleus and notched cartilage. Although according to the basic principles of mastoid surgery, in tubotympanic disease, canal wall up procedure or transcanal tympanoplasty are the preferred choices, in the present study only those patients of tubotympanic disease were included where irreversible mucosal disease involving the tympanomastoid region was confirmed either by preoperative HRCT temporal bone or by peroperative assessment. Therefore, to achieve a dry cavity-less ear, after canal wall down mastoidectomy, cavity obliteration was done with superiorly and inferiorly based flaps of periosteum and subcutaneous tissue. Autologous conchal cartilage was preferred to hydroxyapatite, total ossicular reconstruction prosthesis, PORP or titanium because most of these implants are either costly or commercially not available in this region. None of the relevant literature reported use of notched cartilage with incus interposition in combination with cavity obliteration as their basic surgical technique. 74% patients in the present study achieved postoperative mean ABG < 20 dB, in comparison with the preoperative mean ABG = 29.3 dB, with a *P* < 0.001, which is statistically significant.

In the future, the authors plan to expand this study to a larger sample size with longer follow-up. Also, comparison of this surgical technique with other available techniques using prostheses, in relation to cost analysis and extrusion rate, will be undertaken.

## CONCLUSION

In chronic suppurative ear disease, ossicular chain erosion leads to considerable hearing loss. Necrosis of the long process of the incus is the commonest of all ossicular erosions. Austin group A (M+S+I−) constitutes almost 60% of all ossicular defects. The present series of ossiculoplasty in this group yielded encouraging results. 74% ossiculoplasty patients achieved statistically significant postoperative ABG < 20 dB. Bad prognostic indicators included semicircular canal fistula and granulation around the footplate area. This novel technique of ossiculoplasty with a combination of autologous notched cartilage, incus interposition, and cavity obliteration is physiological, biocompatible, and stable.

## ACKNOWLEDGMENT

A heartfelt thanks to our Department Chair and staff and our patients.

## FUNDING SOURCES

This study is self-funded.

## CONFLICT OF INTEREST

None declared.

## DATA AVAILABILITY STATEMENT

All data supporting the findings of the study are available within the paper and its supplementary information. The complete list of related studies has been cited at relevant places in the text and summarized in Table no. 3, thereby validating the claimed “novelty” of the surgical approach being reported.
